# Mesoporous Polydopamine Nano-Bowls Demonstrate a High Entrapment Efficiency and pH-Responsive Release of Paclitaxel for Suppressing A549 Lung Cancer Cell Proliferation In Vitro

**DOI:** 10.3390/pharmaceutics16121536

**Published:** 2024-12-01

**Authors:** Lindokuhle M. Ngema, Shahinur Acter, Samson A. Adeyemi, Thashree Marimuthu, Mershen Govender, Wilfred Ngwa, Yahya E. Choonara

**Affiliations:** 1 Wits Advanced Drug Delivery Platform, Department of Pharmacy and Pharmacology, School of Therapeutic Sciences, Faculty of Health Sciences, University of the Witwatersrand, 7 York Road, Parktown, Johannesburg 2193, South Africa; lindokuhle.ngema@wits.ac.za (L.M.N.); samson.adeyemi@wits.ac.za (S.A.A.); thashree.marimuthu@wits.ac.za (T.M.); mershen.govender@wits.ac.za (M.G.); 2 Sidney Kimmel Comprehensive Cancer Center, Department of Radiation Oncology & Molecular Radiation Sciences, Johns Hopkins University, Baltimore, MD 21218, USA; sacter1@jh.edu

**Keywords:** mesoporous nanoparticles, polydopamine, paclitaxel, non-small-cell lung carcinoma, anti-proliferation

## Abstract

**Background:** The effectiveness of paclitaxel (PTX) in treating non-small-cell lung carcinoma (NSCLC) is restricted by its poor pharmacokinetic profile and side effects. This limitation stems from the lack of a suitable delivery vector to efficiently target cancer cells. Therefore, there is a critical need to develop an efficient carrier for the optimised delivery of PTX in NSCLC therapy. **Methods:** The present study describes the fabrication of mesoporous polydopamine (mPDA) nano-bowls via an emulsion-induced interfacial anisotropic assembly method, designed for efficient entrapment of PTX and pH-responsive release behaviour. **Results:** The nano-bowls depicted a typical bowl-like shape, with connecting mesoporous channels and a central hollow cavity, allowing optimal loading of PTX. The fabricated nanocarrier system, mPDA-PTX-nb, had a mean hydrodynamic bowl diameter of 200.4 ± 5.2 nm and a surface charge of −39.2 ± 1.3 mV. The entrapment efficiency of PTX within the nano-bowls was found to be 95.7%, with a corresponding release of 85.1% achieved at the acidic pH 5.9 (simulated tumour microenvironment) at 48 h. Drug release was best fitted to the Peppas–Sahlin model, indicating the involvement of both diffusion and relaxation mechanisms. Treatment with mPDA-PTX-nb significantly suppressed A549 lung cancer cell proliferation at 48 and 72 h, resulting in cell viability of 14.0% and 9.3%, respectively, at the highest concentration (100 µg/mL). **Conclusions:** These results highlight the potential of mPDA-PTX-nb as an effective nanocarrier for PTX, promoting enhanced anti-proliferative effects in NSCLC therapy.

## 1. Introduction

Effective treatment of non-small cell lung carcinoma (NSCLC) remains a global challenge which contributes to the continuous increase in the number of lung cancer-related deaths worldwide [1,2]. Approximately 85% of all lung cancer cases are diagnosed as NSCLC.

Sadly, the current chemotherapeutics employed in NSCLC management, including paclitaxel (PTX), and their conventional delivery strategies have been shown to be challenged with poor pharmacokinetic profiles owing to a short half-life, poor water solubility, and non-specificity, causing inadequate delivery of chemotherapeutics to tumours and further resulting in undesired cytotoxicity to healthy cells [3,4]. PTX is one of the first-line anticancer drugs used in NSCLC intervention, and although the drug demonstrates notable efficacy at the onset of intervention, its short half-life and non-specific binding limit its therapeutic potency in NSCLC therapy [5,6].

As such, there is a growing appeal for the design of effective delivery systems for PTX to enhance its therapeutic activity in NSCLC therapy and minimise side effects. Although numerous drug delivery approaches have been explored for various anticancer drugs [7], hydrophobic drugs are difficult to load and entrap using nanocarriers [8]. Essentially, an ideal vehicle for PTX would need to fulfil a set of criteria, such as having high loading capacity and entrapment efficiency for hydrophobic drugs, smaller size (i.e., 10–200 nm) for tumour penetration, and site-specific release for drug deposition only at targeted tumour sites [9]. Achieving a site-specific drug release is crucial in cancer therapy, and by exploiting the characteristic features of the tumour microenvironment (TME), such as its distinct acidic pH profile, environmentally responsive drug carriers are a promising approach for achieving precise drug delivery to tumours [10].

Interestingly, mesoporous polydopamine (mPDA) nanoparticles, formed via self-polymerisation of dopamine, have gained attention as a biocompatible nanocarrier for cancer therapeutics [11,12]. Apart from their biocompatibility, mPDA nanoparticles present other attractive properties, including high drug loading capacity, colloidal stability, robust adhesion to cellular surfaces due to the bowl shape, and a relatively simple fabrication approach [12]. Moreover, mPDA nanoparticles have been shown to exhibit adequate loading capacity for doxorubicin (DOX), for combined chemo and photothermal cancer therapy, with a demonstrated pH-triggered drug release and enhanced in vitro cellular uptake [13,14]. However, not much has been reported on the ability of mPDA nanoparticles to load hydrophobic anti-cancer drugs, particularly PTX, despite its interesting physicochemical features and promising advances in cancer therapy [15].

Previous studies have reported that the anisotropic morphology of mPDA nanoparticles promote enhanced cellular internalisation via caveolin-dependent endocytosis, compared to their spherical counterparts [12,13,16,17]. As such, in the present study, we report on the fabrication of bowl-shaped mPDA nanoparticles (termed nano-bowls) and their potency as a favourable nanocarrier for the effective delivery of PTX, with potential application in NSCLC therapy. Accordingly, appropriate physicochemical characterisation techniques for elucidating medically desired properties such as PTX entrapment efficiency and drug release (including release modelling), particle size and charge, and chemical composition, along with vital in vitro cellular analyses (i.e., antiproliferative activity and biocompatibility) were undertaken to establish the therapeutic merits of the formulated PTX-loaded mPDA nano-bowls (mPDA-PTX-nb) system.

## 2. Materials and Methods

### 2.1. Materials

Dopamine hydrochloride (98%), Pluronic F-127 (12.6 kDa), 1,3,5-trimethylbenzene (TMB, 98%), ammonia solution (NH_4_OH, 28%), phosphate-buffered saline (PBS) tablets, paclitaxel (PTX), tween 80, heparin solution, and 3-(4,5-dimethyldiazol-2-yl)-2,5-diphenyltetrazolium bromide (MTT) Cell Proliferation Kit I were purchased from Sigma-Aldrich (St. Louis, MO, USA). SnakeSkin™ dialysis membrane (3500 MWCO), penicillin-streptomycin, trypsin-EDTA, and 10X PBS were procured from Thermo Fisher Scientific (Waltham, MA, USA). Lung adenocarcinoma cell line (A549), human umbilical vein endothelial cell line (HUVEC), Foetal Bovine Serum (FBS), Dulbecco’s Modified Eagle’s Medium (DMEM), F-12K medium, and endothelial cell growth supplement (ECGS) were procured from ATCC^®^ (Manassas, VA, USA). Reagents used for this study were of analytical reagents (ARs) grade and used as received.

### 2.2. Methods

#### 2.2.1. Fabrication of mPDA Nano-Bowls

A facile emulsion-induced interfacial anisotropic assembly method was employed for the fabrication of mPDA nano-bowls [16]. Briefly, 0.1 g Pluronic F-127 and 0.15 g dopamine hydrochloride were dissolved in 10 mL water–ethanol (1:1). The mixture was stirred at 600 rpm (MST Digital Magnetic Stirrer, VELP Scientifica, Inc., Deer Park, NY, USA) till a clear solution formed. TMB (200 µL) was then added to the solution, which was briefly sonicated for 2 min (Branson Ultrasonics™ SFX150, Fisher Scientific, Waltham, MA, USA). To the resulting emulsion, the dropwise addition of NH_4_OH solution (375 µL) was carried out, and the mixture was further stirred at 600 rpm for 2 h under monitoring. The formulated PDA nano-bowls was centrifuged at 10,000 rpm at 10 °C for 20 min (Eppendorf™ 5424R Microcentrifuge, Fisher Scientific, Waltham, MA, USA). The washing step was repeated 3 times using water–ethanol, with the mPDA nano-bowls collected and lyophilized (FreeZone 4.5 Plus Freeze Dryer Lyophilizer, Labconco Corporation, Kansas City, MO, USA) for further use.

#### 2.2.2. Loading of PTX and Determination of Entrapment Efficiency

An established drug loading approach was implemented [10], with modifications, to load PTX onto the mPDA nano-bowls for exploitation of the nanoparticles as a nanocarrier. Firstly, 30 mg of a lyophilized sample of mPDA nano-bowls was dispersed into 3 mL methanol with subsequent sonication for 2 min (POWERSONIC™ Ultrasonic Cleaner CP360HT, CREST Ultrasonics Corp., Ewing Township, NJ, USA). Secondly, a solution of 3 mg of PTX (10% *w*/*w* of mPDA nano-bowls) in 1 mL of 70% *v*/*v* methanol was prepared, and this was added in a dropwise manner to the mPDA nano-bowl suspension. The mixture was kept under continuous stirring overnight at 200 rpm (MST Digital Magnetic Stirrer, VELP Scientifica, Inc., Deer Park, NY, USA) to allow for adequate drug entrapment.

The resulting suspension was centrifuged at 10,000 rpm (10 °C), for 10 min, with subsequent washes (X3) with 70% methanol for the removal of unentrapped drug. The supernatant, containing unentrapped PTX, was used for determining the drug’s entrapment efficiency (%EE) and the drug loading capacity (%DLC) of mPDA via UV spectrophotometry (Cary™ 50, Varian Inc., Palo Alto, CA, USA) at 230 nm. A standard calibration curve of PTX (2.5–20 µg/mL; 70% methanol; ε = 0.0451) was constructed prior, and the %EE and %DLC was computed using the described Equations (1) and (2), respectively:

(1) $$\%\mathrm{EE}=\frac{D_{l\;}-D_{s}}{D_{l}}\times 100$$

(2) $$\%\mathrm{DLC}=\frac{D_{l\;}-D_{s}}{W_{f}}\times 100$$

where $D_{l}$, $D_{s}$, and $W_{f}$ represent the actual amount of PTX loaded, the amount of PTX in the supernatant (unentrapped), and the total weight of PTX-loaded mPDA nano-bowls, respectively.

#### 2.2.3. Analysis of Chemical Bond Transformations and Structural Properties

Fourier Transform Infrared (FTIR) spectroscopy and powder X-ray diffraction (PXRD) analysis were conducted for the assessment of chemical bond transformations and structural properties of the formulated mPDA nano-bowls, respectively. These were undertaken to confirm the successful formulation of mPDA nano-bowls and further ascertain the presence of PTX within mPDA, thus validating the drug loading procedure. Accordingly, for FTIR analysis, dry powdered samples of blank mPDA nano-bowls, free PTX, and mPDA-PTX-nb were comparatively analysed over 20 scans in a 4000–550 cm^−1^ range at 120 psi pressure, on a PerkinElmer Spectrum 100 FTIR spectrometer (Waltham, MA, USA). Meanwhile, for PXRD analysis, dried samples of mPDA and mPDA-PTX-nb were analysed on a D8 ADVANCE, Bruker XRD Diffract. Suite Eva 2θ-θ (Bruker, MA, USA) set at monochromatic Cu/Kα radiation (λ = 1.54180 Å), 40 kV/100 mA, 2θ range of 5–90°, and scan rate of 10°/min.

#### 2.2.4. Analysis of Particle Size, Surface Charge, and Morphology

The average hydrodynamic size and surface charge (zeta potential) of the synthesized blank mPDA nano-bowls and mPDA-PTX-nb were ascertained from dynamic light scattering (DLS) and phase-analysis light scattering (PALS) measurements, respectively.

A dispersion of the lyophilized samples (~0.5 mg in 2 mL MilliQ water) was analysed in disposable cuvettes on a NanoZS ZetaSizer machine (Malvern Panalytical, Malvern, UK). The in vitro stability of the mPDA-PTX-nb was further assessed by monitoring possible degradation and agglomeration over a 7-day period (in PBS, room temperature), through size and surface charge measurements. Transmission electron and scanning electron microscopy (TEM and SEM) were both utilised to visualise the morphology of blank mPDA nano-bowls on a FEI Talos F200X TEM, ThermoFischer Scientific, Waltham, MA, USA, and ZEISS Electron Microscoep, Carl Zeiss Microscopy Ltd., Cambridge, UK, respectively. An aliquot from the DLS sample was dispensed onto the carbon-coated copper grids for TEM analysis, and on an aluminium specimen stub for SEM analysis, and then allowed to completely dry before the analyses could be performed.

#### 2.2.5. Evaluation of pH-Responsive of In Vitro Release of PTX

The release behaviour of PTX from the mPDA nano-bowls was studied under two different pH conditions. Accordingly, a normal pH buffered solution (physiological pH 7.4) and an acidic pH buffered solution (mimicking lung TME; pH 5.9) were employed, and PTX release kinetics were recorded over 48 h [18]. A sample of mPDA-PTX-nb equivalent to 0.609 mg PTX was dispersed in 2 mL of release media (0.1 M; 0.1% *v/v* tween), appropriately enclosed in a SnakeSkin™ dialysis membrane and placed into the respective buffered release media (30 mL). The samples, in their relevant buffers, were then incubated in a shaking incubator at 37 °C (YIHDER LM-530, YIHDER Co., Ltd., Taipei, Taiwan) and sampled (2 mL) over 48 h at predetermined intervals. The release buffer was replenished after each sampling point with the same amount sampled, to maintain sink condition. The quantity of PTX released was calculated using standard calibration methods using a UV spectrophotometer (Cary™ 50, Varian Inc., Palo Alto, CA, USA) at 230 nm. The dissolution results were modelled as outlined by Abdelgader and co-workers [19].

#### 2.2.6. Assessment of In Vitro Cellular Cytotoxicity on A549 Cancer Cells and HUVEC

The model A549 cell line and HUVEC were procured from ATCC^®^ (Manassas, VA, USA) and appropriately cultured using standard cell culture protocols, for testing the anti-proliferative activity and biocompatibility of the formulated mPDA-PTX-nb, respectively. Briefly, the A549 cells were cultured in DMEM supplemented with 10% FBS and 1% penicillin–streptomycin and incubated at 37 °C and 5% CO_2_. Meanwhile, HUVECs were cultured in F-12K medium supplemented with 10% FBS, 0.1 mg/mL heparin solution, and 0.03 mg/mL ECGS and incubated at 37 °C and 5% CO_2_. The cells were allowed to reach 90% confluence and then seeded at a density of 2.5 × 10^4^ cells/mL in 96-well plates, with each well containing 90 µL cell suspension. The A549 cells were treated in triplicates (*n* = 3) with mPDA-PTX-nb compared to PTX (positive control) at concentrations of 3.125, 6.25, 12.5, 25, 50, and 100 µg/mL for 48 h and 72 h, while HUVECs were only treated with mPDA nano-bowls at similar predetermined concentrations for 72 h, to test the biocompatibility of the nanoparticles. The MTT assay was then carried out using the Cell Proliferation Kit I (Merck, Burlington, MA, USA) as per the manufacturer’s protocol, with the introduction of 10 µL of MTT solution with a subsequent 4 h incubation of the plates, followed by the addition of 100 µL solubilising agent to dissolve the formazan crystals. The plates were incubated overnight at 37 °C, with blank wells containing only the solubilising agent and medium. A multi-modal microplate reader (Victor X3, PerkinElmer, Waltham, MA, USA) was used to read the absorbance at 570 nm (reference = 620 nm) for computing the cell viability (% CV) as shown in Equation (3).

(3) $$\%CV=\frac{A_{test\;}-A_{blank}}{A_{control-}\;A_{blank}}\times 100$$

where $A_{test}$, $A_{blank}$, and $A_{control}$ denote test absorbance, blank absorbance, and control absorbance, respectively, in nm.

#### 2.2.7. Statistical Analysis

Statistical analyses comprising a two-tailed Student’s unpaired *t*-test at a 95% confidence interval were performed on GraphPad Prism (v9.5.1 GraphPad Software Inc., San Diego, CA, SA). A value of *p* < 0.05 was denoted as statistically significant.

## 3. Results and Discussion

### 3.1. Fabrication of mPDA Nano-Bowls and the Assessment of PTX Entrapment Efficiency

Pristine mPDA nano-bowls were successfully synthesised through a simplistic emulsion-induced interface anisotropic assembly method. This method is generally commended for its various merits including time efficiency, high yield, and the ability to produce uniform nanoparticles [13,20]. The synthesis of the mPDA nano-bowls begins with the formation of an interface between the two immiscible liquids, trimethylbenzene (TMB) and water, stabilised by the surfactant Pluronic F127. This stabilisation creates a uniform emulsion, allowing for the growth of island-shaped polydopamine (PDA) seeds at the interface between the TMB and water, creating Pluronic F-127-TMB-PDA composite micelles. Following this, nucleation and anisotropic growth occur, resulting in mesoporous bowl-shaped PDA nanoparticles [16]. Accordingly, the formulation method proved to be apt, resulting in a high yield of stable mPDA nano-bowls.

**Figure 1:**
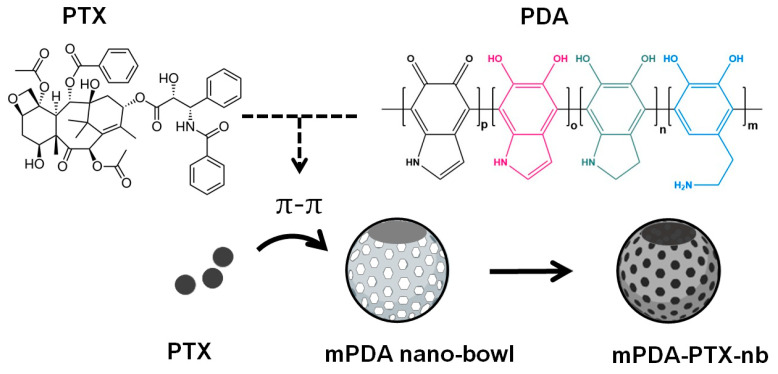
Schematic representation of drug loading into mPDA nano-bowls to formulate mPDA-PTX-nb. The loading of PTX is facilitated through a π-π interaction between the aromatic rings of PTX and mPDA.

In this study, the desired mPDA-PTX-nb system was achieved, with the nano-bowls showing to favour the loading of hydrophobic PTX, with a recorded %EE of 95.7%. The high %EE corresponded to the %DLC of 8.7%, which is close to the actual 10% (*w*/*w*) of PTX that was loaded, indicative of the impeccable capacity of PDA nano-bowls to contain PTX. The aromatic functionalities of both PDA and PTX were the driving force behind the high entrapment of PTX into PDA nano-bowls. It is known that the aromatic systems between unsaturated poly(cyclic) molecules can form non-covalent interactions, known as π-π stacking, which can facilitate the loading of drugs into delivery systems [21,22,23]. Depicted in Figure 1 is the simplistic overview of the drug loading process, with the depiction of the chemical structures of both PTX and PDA, characterised by the aromatic rings, for π-π interaction [23]. Moreover, the high %EE and %DLC obtained could be attributed to the bowl-like shape (presenting with a central cavity) and the mesopores on the nanoparticles, which allow sufficient room for maximal drug loading. While it has been reported that loading hydrophobic drugs into carriers is conventionally challenging without the aid of a hydrophobic polymer, this study successfully loaded PTX without the use of an additional polymer [24]. Moreover, the recorded %EE is comparable to that of a previously formulated nanosystem for delivering PTX which achieved a %EE of 98.5% [10].

### 3.2. Assessment of Particle Size and Surface Charge and Overall Morphology Characterisation

**Figure 2:**
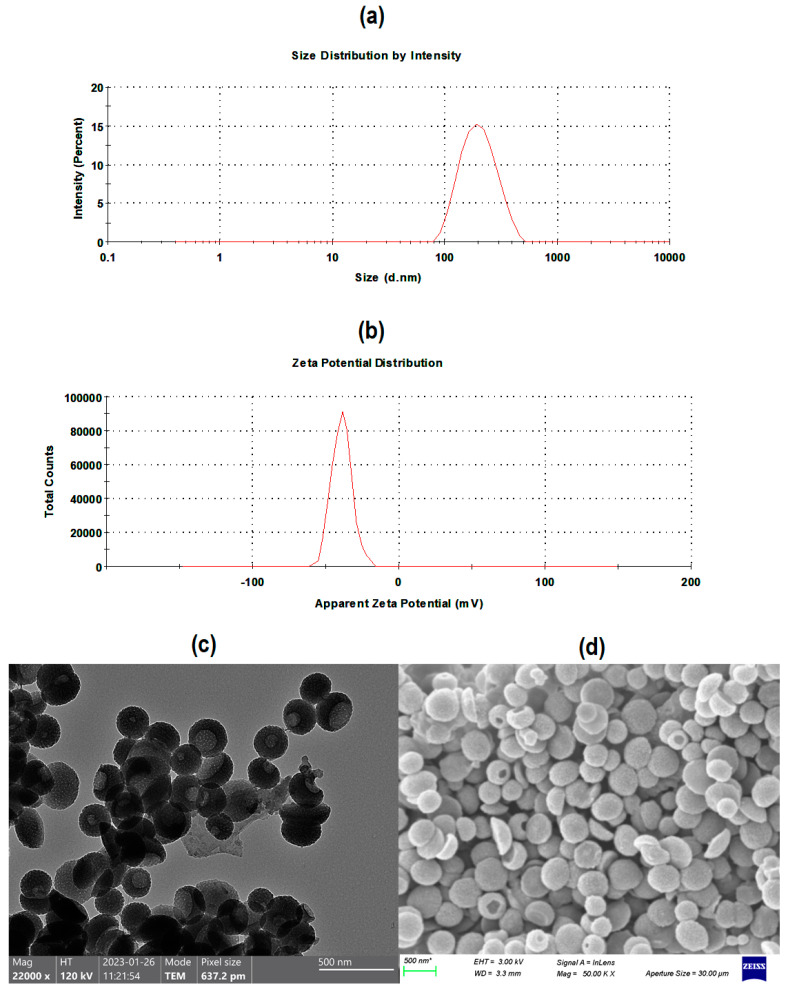
A graphical representation of (**a**) average particle size and (**b**) zeta potential of the synthesized mPDA nano-bowls, with corresponding (**c**) TEM (scale bar = 500 nm; magnification = 22,000×) and (**d**) SEM (scale bar = 500 nm; magnification = 50,000×) images confirming the overall morphology. The size was in an expected nano-diameter range, and the particles exhibited a bowl-like shape with connecting mesoporous channels and a central cavity.

The average particle size and zeta potential (surface charge) of the synthesised pristine mPDA nano-bowls were found to be 192.2 ± 4.6 nm (Figure 2a) and −38.0 ± 1.1 mV, (Figure 2b) respectively, with the size recorded at a polydispersity index (PDI) of 0.19 ± 0.02. The observed size and PDI are indicative of the robustness of the formulation method in controlling the size and the dispersity of the nanoparticles. Essentially, mPDA nano-bowls of sizes 180.0 nm and 267.7 nm, formulated via an emulsion-induced interface anisotropic assembly method, have been reported in the literature, with corresponding PDI readings around 0.20 [13,16]. Fundamentally, both TEM and SEM analyses confirmed the overall morphology of the synthesised mPDA nano-bowls, particularly TEM, which revealed the connecting mesoporous channels (meso-channels) (Figure 2c), and with SEM revealing the central hollow cavity and the bowl-like shape of the nanoparticles (Figure 2d).

Meanwhile, the size and surface charge of mPDA-PTX-nb were found to be 200.4 ± 5.2 nm and −39.2 ± 1.3 mV, respectively, and the PDI at which the size was recorded was 0.24 ± 0.04. The size and PDI exhibited by mPDA-PTX-nb in aqueous solution make this nanocarrier suitable for application in NSCLC therapy, considering that the nanoparticles around 100–200 nm in diameter can penetrate through lung tumours, with less susceptibility to removal by alveolar macrophages [25]. Moreover, the zeta potential (−39.2 ± 1.3 mV) of mPDA-PTX-nb suggests that the nanocarrier presents with colloidal stability, as nanoparticles that exhibit zeta potential values further away from zero (0), ranging between ±30 and ±40 mV in aqueous solutions, are regarded as stable nanoparticle systems [26]. Likewise, mPDA nano-bowls with a zeta potential of −40 mV have been previously reported, proving to be stable when dispersed in water or cell culture medium and PBS [13]. Moreover, mPDA-PTX-nb showed adequate stability over 7 days of storage in PBS at room temperature, as they maintained their average size and surface charge, as presented in Table 1, with corresponding measurement curves provided in Figure S1. Essentially, from the average size and surface charge measurements, it could be deduced that there was no significant particle degradation and agglomeration over time, owing to the colloidal stability of the nano-bowls, as afore mentioned.

### 3.3. Confirmation of Chemical Composition of mPDA Nano-Bowls and PTX and Functional Transformations Towards the Formation of mPDA-PTX-nb

**Figure 3:**
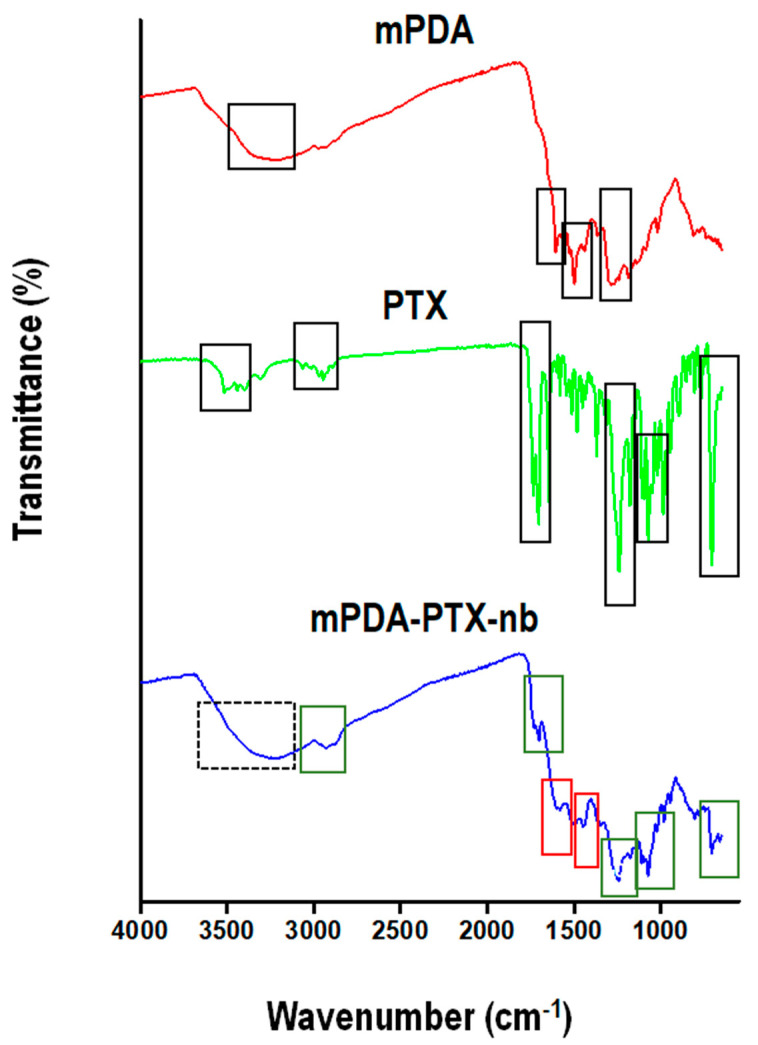
FTIR spectra of blank mPDA nano-bowls, PTX, and mPDA-PTX-nb, showing the characteristic peaks of the materials (marked in solid black). The successful formation of mPDA-PTX-nb is evidenced by the presence of the characteristic peaks of both mPDA nano-bowls (red rectangular markings) and PTX (green rectangular markings) in the spectrum, as well as the overlap of both (black dotted marking).

The chemical composition of blank mPDA nano-bowls, PTX, and the resulting mPDA-PTX-nb were confirmed through the mapping of the characteristic functional groups of these compounds via FTIR spectroscopy (Figure 3). Primarily, FTIR spectroscopy is an ideal technique for robust identification of chemical bonds within moieties [27]. The FTIR spectrum of mPDA nano-bowls exhibited characteristic peaks associated with the primary functional groups of PDA, specifically catechol, amine, quinone, and indole [28]. The peak at 3228 cm^−1^ was attributed to the –OH stretch of the catechol group, and the peak at 1560 cm^−1^ could be attributed to the primary amine (-NH_2_); meanwhile, the peak at 1604 cm^−1^ belonged to the C=O of the quinone group. Additionally, molecular vibration at 1498 cm^−1^ was ascribed to C=C stretching of the indole group, indicative of polymerisation of dopamine to PDA [28]. The FTIR spectrum of PTX displayed characteristic peaks at 3514, 2945, 1704, 1645, and 1240 cm^−1^, corresponding to the –OH stretch, CH stretch, C=O stretching, C=O stretching (amide), and C-O-O bending (ester), respectively (Figure 3). A distinct band at 706 cm^−1^ is indicative of C-H bending vibrations for the three monosubstituted aromatic rings in the structure of PTX [29]. Interestingly, the FTIR spectrum of mPDA-PTX-nb exhibited the characteristic peaks of both mPDA nano-bowls and encapsulated PTX with minor shifts in the bands for PTX, thereby indicating the presence of PTX within the mPDA nano-bowls. Primarily, in the spectrum of mPDA-PTX-nb, the appearance of a broad peak centred around 3214 cm^−1^ (dotted square) most likely corresponded to the overlapping –OH groups of mPDA nano-bowls and PTX. Evidently a downward shift from 2945 to 2927 cm^−1^ for C-H stretching and a slight shift to lower frequencies from 1704 to 1701 cm^−1^ for C=O (ketone) of PTX is indicative of drug loading. In the spectrum of PTX, the peaks being compared are denoted in green rectangular markings.

Lastly, the peaks at 1243 cm^−1^ and 704 cm^−1^ belonging to the ester and aromatic rings of PTX, respectively, could still be observed in the spectrum of mPDA-PTX-nb, which shifted slightly from 1240 cm^−1^ and 706 cm^−1^, respectively. The shift to a lower frequency of the C=C bands in mPDA-PTX-nb indicates modifications in its π-system upon drug loading [30]. In the spectrum of mPDA-PTX-nb, bands at 1579 and 1447 cm^−1^ were slightly upshifted relative to the corresponding–NH and C-H bending observed in the spectrum of mPDA nano-bowls (1560 and 1438 cm^−1^). The peaks belonging to mPDA nano-bowls are highlighted in red rectangular markings. There were no new major peaks observed in the spectrum of mPDA-PTX-nb, as there were no new bonds formed between mPDA nano-bowls and PTX, but only a π-π interaction, as evidenced by minor shifts in the peaks of the groups involved, from higher to lower wavenumbers. Essentially, the non-covalent interactions are not as strong as covalent bonding, which results in the shift to lower wavenumbers, as opposed to stronger bonds which would absorb at higher wavenumbers [31].

### 3.4. Assessment of Chemical and Structural Properties of mPDA and mPDA-PTX-nb

**Figure 4:**
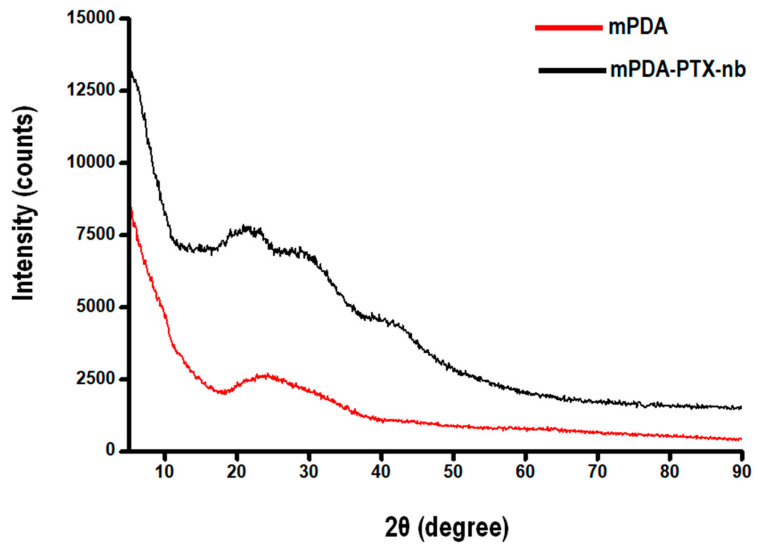
PXRD patterns of mPDA and mPDA-PTX-nb elucidating the π-π stacking assembly and the amorphous state of the formulated nano-bowls.

The chemical fingerprint of blank mPDA nano-bowls and the formulated mPDA-PTX-nb was ascertained through the tracing of the π-π stacking assemblies, envisaged from the π-π interactions between PDA and π-electron-rich TMB to form mPDA, as well as the π-π interactions between PTX and mPDA, forming mPDA-PTX-nb. The diffraction patterns are presented in Figure 4 below. Accordingly, a diffraction pattern from mPDA showing a broad peak at 2θ = 23° was obtained, which corresponds to the π-stacking in polydopamine that emanates from the orderly assembly of dopamine oligomers [23]. Similarly, a comparative PXRD pattern of mPDA nanoparticles has been reported by Chen et al., 2016 [23]. Meanwhile, a diffraction pattern of mPDA-PTX-nb also showed a broad peak at 2θ = 23° with an increased intensity than that of blank mPDA. The peak at 2θ = 23° corresponds to the π-π stacking assembly as observed from the mPDA pattern, with the increase in intensity signifying the multiplicity of the π-π plane, attributed to the additional π-π interaction between mPDA and PTX. This correlates with the FTIR data and further confirms that the loading of PTX onto mPDA nano-bowls is indeed via a π-π interaction, as previously reported [13,21,22]. Moreover, the observed peaks further confirmed the amorphous state of mPDA [23], and it could be concluded that PTX was encapsulated in the amorphous state, since no apparent PTX peaks could be observed from the diffraction pattern of mPDA-PTX-nb [32].

### 3.5. Assessment of In Vitro pH-Responsive Release of PTX from mPDA-PTX-nb

**Figure 5:**
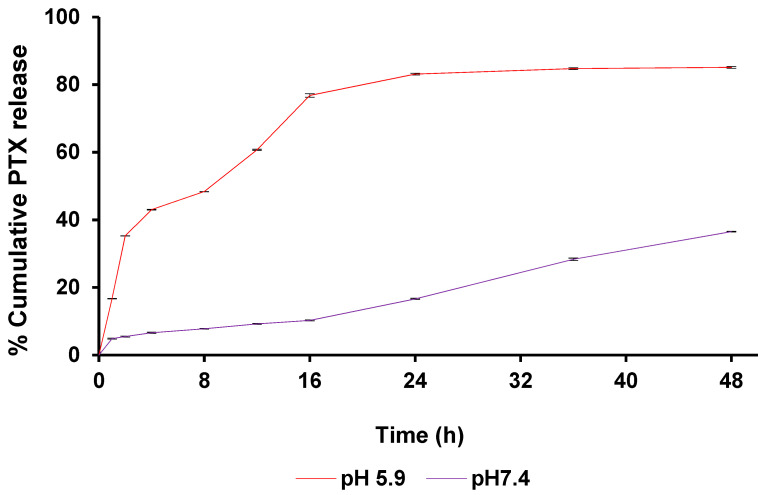
Cumulative in vitro release of PTX at pH 5.9 and pH 7.4 over 48 h. Optimal drug release of 85.1% (48 h) was recorded at acidic pH 5.9, and only 36.5% (48 h) was recorded at the physiological pH (7.4). The data are expressed as mean ± standard deviation (SD) with a sample size of *n* = 3.

To investigate the influence of pH on the release of PTX from the mPDA nanocarrier, in vitro drug release studies were conducted. Accordingly, presented in Figure 5 is the cumulative release profile of PTX at an acidic pH of 5.9 and physiological pH of 7.4 over 48 h. The release of PTX at pH 5.9, which simulates the tumour microenvironment (TME), was significantly higher than the release observed at physiological pH 7.4. This indicates that the acidic conditions favour the release of PTX. Essentially, a sustained release of PTX could be observed over time at pH 5.9, with an optimal release of 85.1% reached at 48 h. This elevated and sustained release of PTX is attributed to the disruption of π-π stacking/interaction between mPDA nano-bowls and PTX over time, as the amine group of PDA gets protonated in an acidic environment [13,33]. A similar drug release profile has been reported in the literature for docetaxel, where over 80% of the drug was released from PDA nano-bowls at an acidic pH of 5.0–6.0 [13,15,33].

Meanwhile, the release of PTX at pH 7.4 was relatively lower, achieving a maximum release of 36.5% after 48 h. A previous study has also reported a comparable release performance of PTX from poly (ethylene) glycol-modified PDA nanoparticles, where about only 20% of PTX was released from the nanocarrier in PBS over 24 h [34]. In the present study, the limited release of PTX recorded at pH 7.4, in contrast to the higher release recorded at pH 5.9, signifies the ability of mPDA nano-bowls to facilitate a controlled and site-specific release of PTX, which is crucial in targeted cancer therapy for enhanced therapeutic outcomes and limited side effects. Essentially, the pH-stimulated drug release performance observed in the present study suggests that our nanocarrier can potentially deliver PTX directly into tumours, with adequate PTX release triggered by the acidic pH of the TME, while ensuring minimal unwanted drug release into healthy cells.

Dissolution data modelling of the obtained drug release profiles (Table 2) noted different release mechanisms employed at the pHs evaluated. Under both lung tumour microenvironment (TME) conditions (pH 5.9) and normal physiological conditions (pH 7.4), the release of PTX from mPDA-PTX-nb was best described by the Peppas–Sahlin model and subsequently the Korsmeyer–Peppas model. This suggests that the release mechanism involves both diffusional and relaxation behaviour [19]. This was confirmed in the disparity between the “*n*” value in the Korsmeyer–Peppas model (0.322) and the “m” value in the Peppas–Sahlin model (0.563) at pH 5.9, noting relaxation of the system, while the ‘n’ value from the Korsmeyer–Peppas model further noted a release corresponding to Fickian diffusion [35,36]. Additionally, the large difference in K_1_ and K_2_ in the Peppas–Sahlin model confirmed that the mechanism of PTX release displayed Fickian kinetics at pH 5.9. At pH 7.4, the high ‘n’ value in the Korsmeyer–Peppas model (0.909) was indicative of super case II transport, indicating that drug release at pH 7.4 was not dependent primarily on diffusion but due to compressive stress on the system leading to drug release [36,37]. These results, therefore, validated the pH-responsive nature of the mPDA-PTX-nb system at both lung TME and normal physiological conditions.

### 3.6. Assessment of In Vitro Anti-Proliferative Activity of mPDA-PTX-nb on A549 Lung Adenocarcinoma Cells and Biocompatibility of mPDA Nano-Bowls on HUVEC

**Figure 6:**
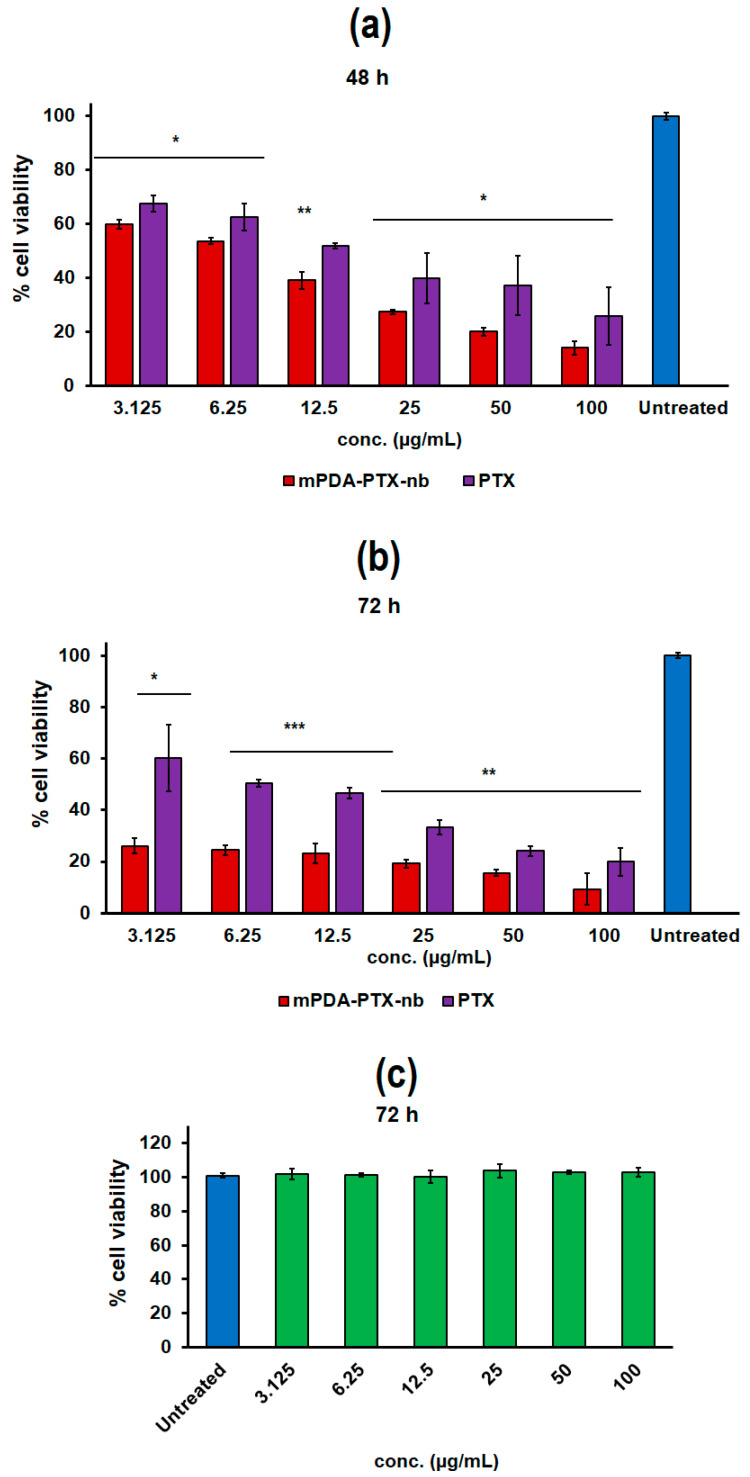
Graph showing presentation of cell viability of A549 cells after (**a**) 48 h and (**b**) 72 h of treatment with mPDA-PTX-nb and PTX (control) at varying concentrations and (**c**) cell viability of HUVEC after incubation with mPDA nano-bowls for 72 h. The data are expressed as mean ± standard deviation (SD) with a sample size of *n* = 3, where * is *p* < 0.05, ** *p* < 0.01, and *** *p* < 0.001, indicating statistical significance between mPDA-PTX-nb and PTX.

The anti-proliferative activity of the formulated mPDA-PTX-nb was aptly deduced in vitro from the % cell viability of A549 cells after 48 h and 72 h of treatment at varying concentrations (Figure 6a,b). Primarily, the cell viability was found to be dose-dependent, with a lower cell viability with increasing concentrations. Briefly, at 48 h, mPDA-PTX-nb resulted in a significantly lower cell viability compared to PTX (control drug), with the maximum treatment dose of mPDA-PTX-nb (100 µg/mL) yielding a cell viability of 14.0% compared to 25.8% recorded from PTX at the same treatment dose. Likewise, the lowest concentration of mPDA-PTX-nb (3.125 µg/mL) yielded a significantly lower cell viability of 59.9%, compared to the 70.5% cell viability recorded from the PTX treatment at an equivalent concentration.

The cell viability at 72 h was significantly reduced, with only 9.3% viable cells recorded from the treatment with the highest concentration (100 µg/mL) of mPDA-PTX-nb, and 26.1% cell viability from the lowest treatment dose (3.125 µg/mL). These were significantly lower than the cell viability recorded from treatment with pristine PTX at equivalent concentrations. The performance of mPDA-PTX-nb thus evidenced the ability of the nanocarrier to improve the bioactivity of PTX, resulting in enhanced inhibition of proliferation of A549 cells. Essentially, the enhanced anti-proliferative activity observed from mPDA-PTX-nb is attributed to the sustained release of PTX, which actively suppresses the proliferation of A549 cells over time, as opposed to the pristine drug which may only elicit effects at the onset of treatment, but limited activity over time, particularly at extended periods like 48 h and 72 h. Likewise, the enhanced anti-proliferative activity demonstrated by mPDA-PTX-nb is further linked to the morphology of the nanoparticles, which enable robust cellular internalisation via endocytosis, as previously reported in our preliminary studies by Acter et al., [16,17]. It is reported that the shape and size of mPDA nanoparticles are known to play a crucial role in their cellular uptake efficiency. These factors also significantly impact the nanoparticles’ intracellular trafficking [17].

Essentially, the anisotropic bowl-like shape of mPDA nanoparticles with increased surface area allow for strong cellular adherence and caveolin-mediated endocytosis [16,17]. Accordingly, the bowl-shaped mPDA nanoparticles get widely distributed within the cells, with less aggregation, in contrast to their spherical counterparts [17]. This is illustrated in Figure S2. Additionally, the biocompatibility of the mPDA nano-bowls was confirmed after 72 h, and the results are presented in Figure 6c. Biocompatibility is one of the crucial characteristics that a drug delivery system must possess, particularly in cancer therapy, in order to eliminate any unwanted cytotoxicity to healthy cells when the drug is in transit [38]. Interestingly, mPDA nano-bowls did not show any signs of toxicity to HUVEC, with different concentrations all yielding a cell viability comparable to the untreated control, indicative of undisturbed cell proliferation. Endothelial cells are essential components of blood vessels and play a vital role in supporting the proliferation of healthy cells [39]. Therefore, HUVECs served as an ideal model system for assessing the effects of mPDA nano-bowls on normal cell function.

## 4. Conclusions

In this study, bowl-shaped mesoporous PDA nanoparticles were efficiently fabricated using the emulsion-induced interfacial anisotropic assembly method. The synthesized mPDA nano-bowls demonstrated the desired aptness for loading PTX, a hydrophobic anticancer drug, yielding a potent PTX delivery nanosystem, mPDA-PTX-nb, with a pH-stimulated drug release and a significant growth inhibition of lung adenocarcinoma cells in vitro. These attributes, in addition to the known robust cellular adherence and uptake provided by the anisotropic morphology of mPDA nanoparticles, make mPDA-PTX-nb more efficient as a PTX carrier, relative to spherical nanoparticles. Given the fact that it is generally challenging to efficiently load hydrophobic drugs and control their release, the high entrapment efficiency and the pH-stimulated release of PTX demonstrated by mPDA nano-bowls is a testament of the nanoparticles’ suitability to function as an alternative avenue for the effective loading of PTX for application in treating NSCLC. The mPDA-PTX-nb did not only enhance the activity of PTX, but further exhibited desirable physicochemical properties, such as aqueous stability and smaller size, which are generally favourable for the design of effective lung cancer nanomedicines, for evading physiological barriers and allowing adequate tumour penetration. Further in vivo studies can ascertain the efficacy of mPDA-PTX-nb in suppressing tumour growth.

## Figures and Tables

**Table 1:** Average particle size, PDI, and zeta potential measurements for stability assessment over 7 days. Data presented as mean ± standard deviation (*n* = 3).

	Average Size (nm)	Polydispersity Index (PDI)	Zeta Potential (mV)
Day 01	200.10 ± 0.30	0.29 ± 0.03	−38.90 ± 0.36
Day 03	199.97 ± 1.23	0.29 ± 0.02	−39.00 ± 1.05
Day 07	199.27 ± 0.21	0.28 ± 0.04	−38.33 ± 0.55

**Table 2:** Modelling and release kinetics of PTX release from mPDA-PTX-nb at pH 5.9 and pH 7.4.

pH	Zero Order		First Order		Higuchi		Korsmeyer–Peppas			Peppas–Sahlin		
	R^2^	K_0_	R^2^	K_1_	R^2^	K_H_	R^2^	*n*	K_KP_	R^2^	K_1_	K_2_
pH 5.9	0.1223	2.514	0.8803	0.091	0.8471	15.297	0.9512	0.322	27.005	0.9804	21.491	−1.342
pH 7.4	0.9528	0.762	0.9483	0.009	0.8548	4.142	0.9559	0.909	1.052	0.9661	1.370	0.143

## Data Availability

Additional data supporting this study can be found in the Supplementary Materials, as well as upon request from the corresponding author.

## Supplementary Materials

The following supporting information can be downloaded at https://www.mdpi.com/article/10.3390/pharmaceutics16121536/s1: Figure S1: Size and zeta potential measurements for stability testing over 7 days in PBS at room temperature. The raw DLS and PALS readings are recorded in triplicates (*n* = 3), from which the mean and standard deviation are computed. The representative frequency curves show the size (**top**) and zeta potential (**bottom**) distribution at day 01, 03, and 07. Figure S2: Graphical illustration of the favoured cellular adherence and caveolin-mediated endocytosis of bowl-shaped mesoporous polydopamine (mPDA) nanoparticles in comparison to their spherical counterparts. The anisotropic bowl-like shape of mPDA nanoparticles with increased surface area allow for strong cellular.
