# Supplementary material for: Mesoporous Polydopamine Nano-Bowls Demonstrate a High Entrapment Efficiency and pH-Responsive Release of Paclitaxel for Suppressing A549 Lung Cancer Cell Proliferation In Vitro
Source: Pharmaceutics. 2024 Dec 1;16(12):1536. doi: 10.3390/pharmaceutics16121536 (PMC11676260; doi:10.3390/pharmaceutics16121536)
Supplement: Supplementary file 1 [file pharmaceutics-16-01536-s001.zip › pharmaceutics-3313173-supplementary.pdf]

Supplementary Information

Mesoporous polydopamine nano-bowls demonstrate a high entrapment efficiency and pH-responsive release of paclitaxel for suppressing A549 lung cancer cell proliferation *in vitro*

Lindokuhle M. Ngema [1,2], Shahinur Acter [2], Samson A. Adeyemi [1], Thashree Marimuthu [1], Mershen Govender [1], Wilfred Ngwa [2,*] and Yahya E. Choonara [1,*]

DLS & PALS readings (n=3)

14	Size	PDA-PTX_Day01	1	12 November 2024 01:21:22 PM	25.0	200.1	0.303				0.00	Nano series
15	Size	PDA-PTX_Day01	2	12 November 2024 01:23:25 PM	25.0	200.4	0.311				0.00	Nano series
16	Size	PDA-PTX_Day01	3	12 November 2024 01:25:28 PM	25.0	199.8	0.251				0.00	Nano series
17	Zeta	PDA-PTX_Day01	1	12 November 2024 01:27:35 PM	25.0			-39.3	-1.849	0.00573	0.00	Nano series
18	Zeta	PDA-PTX_Day01	2	12 November 2024 01:30:42 PM	25.0			-38.8	-1.944	0.0206	0.00	Nano series
19	Zeta	PDA-PTX_Day01	3	12 November 2024 01:31:22 PM	25.0			-38.6	-2.105	0.00558	0.00	Nano series
20	Size	PDA-PTX_Day03	1	15 November 2024 01:40:46 PM	25.0	198.6	0.262				0.00	Nano series
21	Size	PDA-PTX_Day03	2	15 November 2024 01:43:09 PM	25.0	201.0	0.304				0.00	Nano series
22	Size	PDA-PTX_Day03	3	15 November 2024 01:45:32 PM	25.0	200.3	0.300				0.00	Nano series
23	Zeta	PDA-PTX_Day03	1	15 November 2024 01:48:44 PM	25.0			-37.9	-1.973	0.00514	0.00	Nano series
24	Zeta	PDA-PTX_Day03	2	15 November 2024 01:51:45 PM	25.0			-39.1	-1.834	0.00841	0.00	Nano series
25	Zeta	PDA-PTX_Day03	3	15 November 2024 01:52:25 PM	25.0			-40.0	-3.845	0.00521	0.00	Nano series
26	Size	PDA-PTX_Day07	1	19 November 2024 02:22:04 PM	25.0	199.1	0.247				0.00	Nano series
27	Size	PDA-PTX_Day07	2	19 November 2024 02:25:08 PM	25.0	199.5	0.319				0.00	Nano series
28	Size	PDA-PTX_Day07	3	19 November 2024 02:28:13 PM	25.0	199.2	0.272				0.00	Nano series
29	Zeta	PDA-PTX_Day07	1	19 November 2024 02:32:47 PM	25.0			-38.9	-1.897	0.00887	0.00	Nano series
30	Zeta	PDA-PTX_Day07	2	19 November 2024 02:35:48 PM	25.0			-38.3	-1.560	0.0123	0.00	Nano series
31	Zeta	PDA-PTX_Day07	3	19 November 2024 02:36:28 PM	25.0			-37.8	-1.417	0.00886	0.00	Nano series

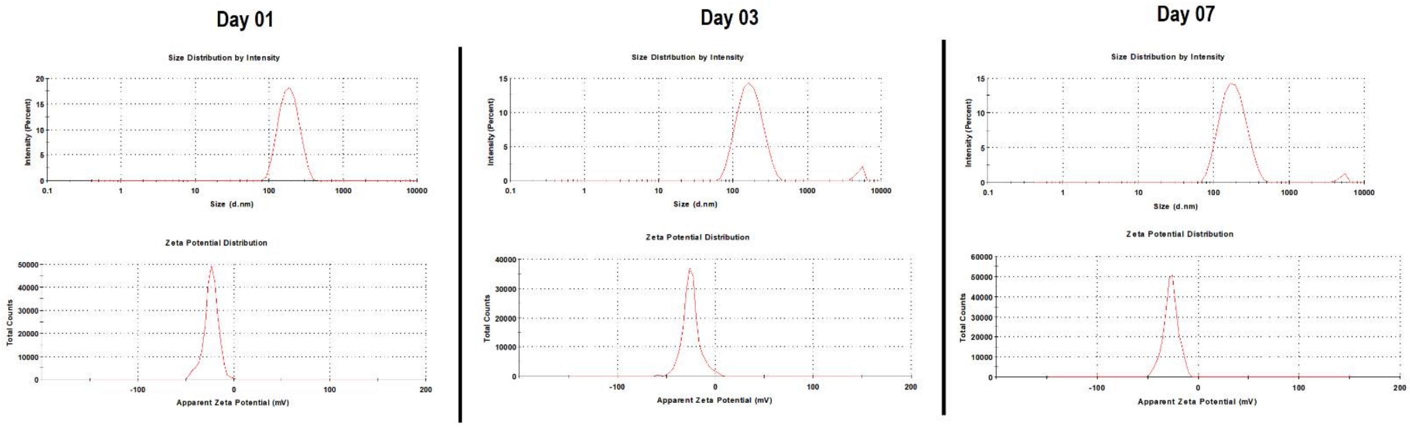

Figure S1. Size and zeta potential measurements for stability testing over 7 days in PBS at room temperature. The raw DLS and PALS readings are recorded in triplicates (n=3), from which the mean and standard deviation are computed. The representative frequency curves show the size (**top**) and zeta potential (**bottom**) distribution at day 01, 03, and 07.

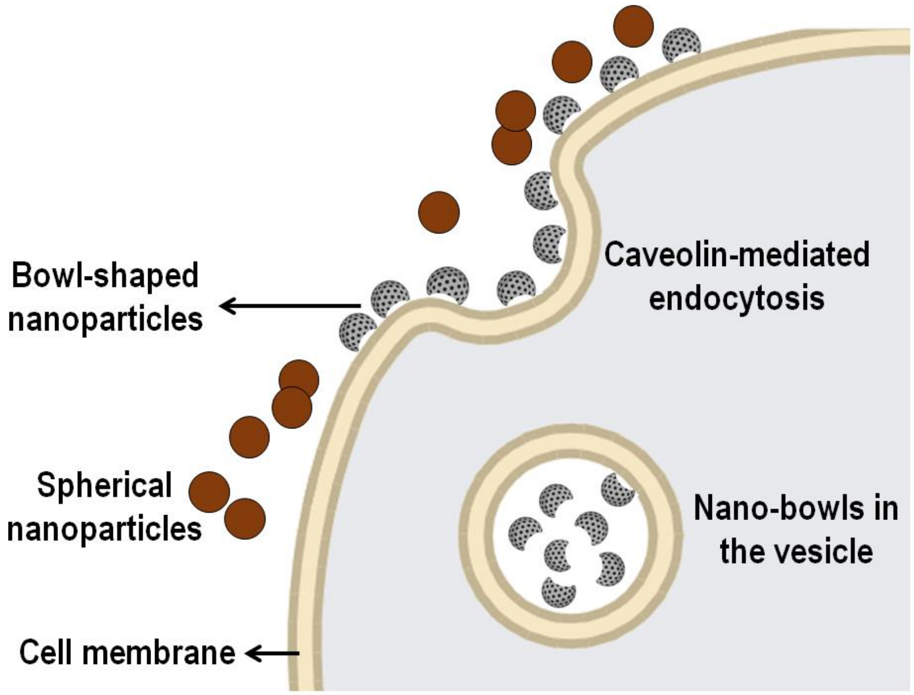

Figure S2. Graphical illustration of the favoured cellular adherence and caveolin-mediated endocytosis of bowl-shaped mesoporous polydopamine (mPDA) nanoparticles in comparison to their spherical counterparts. The anisotropic bowl-like shape of mPDA nanoparticles with increased surface area allow for strong cellular adherence and caveolin-mediated endocytosis, culminating in enhanced cellular uptake.
